# In Search of the Missing Links Between Economic Insecurity and Political Protest: Why Does Neoliberalism Evoke Identity Politics Instead of Class Interests?

**DOI:** 10.3389/fsoc.2020.00028

**Published:** 2020-04-29

**Authors:** Juha Siltala

**Affiliations:** ^1^Department of Philosophy, History and Art Studies, University of Helsinki, Helsinki, Finland; ^2^Department of Philosophy, History, Culture and Art Studies, Faculty of Arts, University of Helsinki, Helsinki, Finland

**Keywords:** status anxiety, middle class, attachment to groups, coalition formation, identity politics

## Abstract

The prospect of the social backsliding of middle-class groups in western countries has not benefited the left but fueled right-wing populism. This article examines mediating and moderating factors between economic threat and political choices. The shift of liberals toward conservatism and the activation of passive authoritarians explain sudden changes more than dispositional factors. Attachment to groups under stress activates coalitional mindsets, and coalitional competition for scarce resources matches the conservative propensity to detect threats from outgroups. Risk-averse right-wing authoritarians should recoil from social-dominance oriented risk-takers but they follow winners despite their mutual differences concerning family values. Authoritarian aggression unites RWA and SDO, but politically passive right-wing authoritarians can also follow their economic interests, when these are not entangled with cultural values. Right-wing populists have been able to compensate economic insecurity with epistemic security. Identity politics supports the coherence of right-wing populist parties but divides leftist/liberal groups due to intersectional competition for victimhood.

## Mysterious Links

The rise of right-wing populisms during the long recession/stagnation following the implosion of global finance in 2008 is spurring researchers to reconsider their theories of motivation. Economic losses, real and perceived, and persistent insecurity, have not led to alternative economic policies. Traditional class parties have been shrinking, while a new Manichean political division is leaning on cultural values. The goal of this article is to determine how economic anxieties are mediated into boosting status through collective identifications.

Stressful economic changes can be taken as a given on the basis of the literature on economic history and politics in the U. S. and in Western Europe since WWII, and especially concerning the contrast between the “golden three decades” after the war and the last three decades of neoliberal financialization. The age of wars and extremisms was followed by an era of domesticated capitalism: labor-related income rose in pace with growth and productivity, full employment was attained and risks were collectivized by means of social insurance and welfare services. This long cycle based on the mass production of consumer durables, cheap raw material and energy, the communist isolation of most of Asia from competition, and, last but not least, the ideological competition that compelled employers to pay decent wages to pre-empt socialism came to an end in the 1970s through an overproduction crisis and decreasing profits. Since then, collective bargaining and trade unions have been diminished under corporate pressure, and the freedom of capital movement has rendered national politics into mere adaption to global competition. Welfare services have retrenched and individuals have shouldered more responsibility for their employability and health. Incomes have polarized after the long compression, especially through the difference in taxation of capital income and labor income (Castel, 1996; Brenner, 2006; Brynjolfsson and McAfee, 2014; Piketty, 2014; Bivens and Mishel, 2015; Nachtwey, 2016; Cohen, 2017; Goodman and Soble, 2017; Lakner, 2017; Tyson and Spence, 2017; Weil, 2017; Temin, 2018; Tooze, 2018). The financialized economy is no longer reinvigorated through job creation and wages but by boosting asset and real estate values with “quantitative easing.”

In defense of global exchange as a positive-sum game against populisms, many researchers have emphasized that Western countries have not been immiserated, even though Asian middle classes have been the biggest winners, along with the global top 1%. Automation and global cheap labor have not destroyed the middle classes: the upper middle classes have been growing more rapidly even as the lower ones have contracted. Social mobility still occurs and people still reach the upper tiers of income (Hirschl and Rank, 2015; Milanović, 2016; Rose, 2016; Pinker, 2018).

A buoyant “average” does not, however, remove the sense of relative loss for individuals, regions, and groups. Income volatility due to job changes has increased. Unpredictable work makes debt service difficult (Dynan, 2010; Cooper, 2014). In the U. S., even college-level salaries stagnated during the 2000s (Inglehart and Norris, 2017). The USA overcame the recession as soon as 2010 but even the highest wages reached their pre-crisis level as late as in 2015, despite full employment (Schmitt et al., 2018). This trend of decoupling labor income from growth and rising asset prices has been obvious in Germany and Finland, as well. Younger generations no longer believe in their chances to achieve and surpass the standard of living of their parents (Pew Charitable Trusts, 2011; Pew Research Center/Global Attitudes and Trends, 2014; Luttrell and McGrath, 2015; Deal and Levenson, 2016; Kinnunen and Mäki-Fränti, 2016; Twenge et al., 2016; Brooks, 2017; Wichter, 2017). People mostly concerned about falling backward are those low enough but still with a significant measure of status to defend (Kuziemko et al., 2014).

It is better to ignore the short-term booms and busts and take this long-term increase in risks and shortening of expectations as the possible explaining factor for the crisis of the political systems and the pursuit of self-esteem through identity politics.

The horizon of expectations is linked to one's sense of agency. Antonovsky (1979) defines the sense of coherence, crucial to coping, as a “feeling of confidence that one's environment is predictable and that things will work out as well as can reasonably be expected.” It's a mixture of optimism and control. People can accept inequality, if they can hope to improve their lot by work (Kelley and Evans, 2016). Hope means that one is able to project oneself into the future (Cloninger, 2011). The sense control, certainty and meaning have been acknowledged as crucial to stabilizing the human psyche (Hogg and Adelman, 2013; Kay and Eibach, 2013).

The loss of predictable careers, benefits, retirement plans, and the diminishing expectations for one's children have been shattering people's basic trust in the rules (Wilkinson and Pickett, 2018). The L-HPA system, the stress reaction mechanism of individuals, is not equally influenced by all stressors, but primarily by uncontrollable stressors that have social-evaluative aspects (Dickerson and Kemeny, 2004).

The self-destructive behavior of workers studied by Anne Case and Angus Deaton reflect hopelessness, but even well-to-do white collars in Silicon Valley, for example, are preoccupied by fears of losing their positions (Cooper, 2014; Case and Deaton, 2015a,b; Pew Research Center, Social and Demographic Trends, 2015).

The present study evaluates the findings of political psychology on the relationship between economic threats and the turn away from class-based identifications.

I seek the explanation for the growing identity populism from the following starting points.

- Economic stress obviously activates latent authoritarian tendencies and can render even liberals less tolerant.
- Economic disruptions can fuel nationalist populism, because belonging to an identity group can restore one's sense of control and coherence by means of attachment.
- Disruptive winners (those oriented to social dominance, SDO) and those who seek refuge from status threats (right-wing authoritarians, RWA) could be imagined as antagonists but obviously they complete each other.

The first one deals connects authoritarian reactions with the historical situation summarized above. The second one takes into account such ultimate dispositions as coalition formation and self-balancing by means of group attachment, likewise triggered by threat. The third one problematizes the mutual attraction between disruptive economic liberals and risk-averse cultural conservatives and why these can form identity-based tribes, using the tools provided by the first two paragraphs to explain the connection. Finally, I discuss why identity politics serves better right-wing movements than the left.

## Liberals Reacting Like Conservatives and Conservatives Becoming More Conservative

False consciousness has since the 1930s served as a stock explanation for deviations from economic self-interests (Jost and van der Toorn, 2012). Authoritarian personalities identify themselves with the aggressor and blame the weak. However, this explanation does take individual dispositions as causal factors ignoring situational reactions. Furthermore, it does not consider the improvement in child-rearing since the 1960s and the impact of the long-lasting economic security on younger generations who can hardly be classified as authoritarians (Inglehart and Norris, 2017).

The mass support suddenly elicited by a Donald Trump cannot be explained by the dispositional authoritarianism of voters, since the quasi-permanent disposition toward authoritarian solutions can hardly change between two presidential elections. Since individual differences in negativity bias may remain stable over time (Norris et al., 2011), the rise of right-wing movements during the 1930s and since 2007 might be understood as situational authoritarian reactions triggered by experiences of disorder and injustice. When disruptions are interpreted as a culture war and not as a class struggle, right-wing populism will result: authoritarian reactions attempt to restore existential security and cognitive order.

The threats evoking RWA reactions are nowadays accepted as being external, and are no longer viewed as internal tensions, as was the case in early theories on the authoritarian personality. Yet they are moderated by the dispositional frame of RWA: social instability, criminality, deviance, and unemployment appear primarily as disorder to potentially authoritarians (Duckitt, 2013; Schaffer and Duckitt, 2013). According to Stenner (2005), latently authoritarian people are likely to remain tolerant when they do not feel themselves or their way of life to be threatened. White hetero men in Western countries obviously experience that the direction of history threatens their way of life right now. Doty et al. (1991) differentiated dispositional from situational authoritarians. Societal stress activates the authoritarian potential among those who already are dormant authoritarians (Feldman and Stenner, 1997; McCann, 2008; Duckitt, 2013). Unexpected, ambiguous, disorderly stimuli generate more sympathetic system responses in conservatives (Hibbing et al., 2014a). When people have to compete for scarce resources, they become more materialistic and violent, less trustful and less tolerant and support more authoritarian leaders: they concentrate on seizing immediate rewards at the cost of others and neglect thereby not only the development of their cooperative capacities but also any long-term care of themselves and their offspring. Their horizon and strategy have shrunken. Stability based on externalization as opposed to neuroticism seems to be linked with system justification in authoritarian regimes and with right-wing economic attitudes in democratic systems (Fatke, 2017, p. 887, 895).

Threats do not only activate dormant authoritarians but they can also make non-authoritarians move toward intolerance (Heatherington and Suhay, 2011). The needs for control and safety do not basically differentiate liberals and conservatives. Losses increase authoritarianism and decrease empathy in both groups. Economic upheavals have been associated with increases in F-scale scores (loyalty oaths, suppression of erotica, dominant cartoon characters; Sales, 1973; Sales and Friend, 1973; Doty et al., 1991). Experimental studies with manipulated threats and longitudinal studies at national levels have shown higher RWA in people who perceive themselves to be under threat (Doty et al., 1991; Duckitt and Fischer, 2003; Jugert and Duckitt, 2009). Perceived loss of control motivates those low in authoritarianism to resort to authoritarian attitudes in order to regain a sense of control (Mirisola et al., 2014). Social liberals, who are distinguished from risk-averse conscientious conservatives by their openness (Fatke, 2017, p. 884), can lose some of their adventurousness when their basic security is threatened. Mortality reminders and the criticism of one's country by foreigners can cause liberals to think like conservatives: the great aggressively against the challengers of their worldviews (McGregor et al., 1998; Nail et al., 2009; Jonas and Fritsche, 2013).

When threatened, both liberals and conservatives seek confirmation of their opinions from the like-minded, select information, and reject contradictory evidence (Skitka et al., 2002; Castano et al., 2011; Proulx et al., 2012; Chambers et al., 2013; Crawford et al., 2013; Brandt et al., 2014).

Reminded of their basic values under threat, liberals can cling to liberal values (including intergroup fairness) even more vehemently rather than moving to the right (Jonas and Fritsche, 2013). If the propensity to alleviate stress under threat by evoking ingroup values crosses the party divide but does not alter the values invoked, then the demand for group conformism may be the factor that alters under threat. The impact of a tribal mindset and identity-protective cognition on liberals has been speculated about in regard to the heated emphasis on identity politics: ethnic, gender, and cultural animosities seem to be triggered more easily than class-based solidarity across identity boundaries (Mann, 1993/2013; Lilla, 2016; Nagle, 2017, p 43–44, 68–85; Pinker, 2018, p. 356–374). Convay et al. have tried to disentangle authoritarianism and ideology by showing, how leftist and liberal people let values override information, close their minds and express both rigidity (prejudice, dogmatism, and strong attitudes) and authoritarian punitiveness depending on content domain (Convay et al., 2018; Dervin, 2018). If the equation between rigid use of symbols and conservatism would be dissolved, authoritarianism and conservatism could no longer be used as synonyms in political psychology (see Jost et al., 2003).

Cultural closure attempts to maintain predictability in the social world (Thorisdottir and Jost, 2011). Seen from the point of view of personal coherence, people adhering to cultural closure are overstretched by external forces and try to restore a sense of control by symbolic means. When people's sense of control is threatened, they prefer social hierarchy (Friesen et al., 2014), as if it offered a way of proxy control. Cross-country comparison indicates how authoritarianism buffers threatened well-being (Landau et al., 2015; Onraet et al., 2016).

Individual dispositions toward conservatism and liberalism can be taken as mediating and moderating factors between economic threat (cause) and political movements (reaction), not as ultimate causes of political movements. Economic stress can be mitigated by increasing symbolic control and confirming one's identity. This should imply that status politics is less in demand, if everyday life becomes more predictable.

## Belonging To Groups As Compensatory Control

Even the illusion of control can provide health benefits while a loss of control can elicit aggression (Baumeister, 2005, p. 93–103). Surveys have shown that people experience all that they can personally influence as positive (family, hobbies, the content of their work), whereas bigger issues (politics, the economy, the world) beyond their control appear as lost or corrupted (Whitman, 1998). Extremism offers a sense of compensatory control. When people feel uncertain, they may radicalize to restore their sense of control (Hogg and Adelman, 2013; Kay and Eibach, 2013. Cf. criticism by Safra et al., 2018). In the U. S., most perpetrators of ideologically motivated crimes have earlier experienced economic and social losses (Kruglanski et al., 2014; Jasko et al., 2017). Ideologies justify individual quests for self-esteem and significance: devotion to larger issues restores self-worth, shaken by rejection, loss of control and injustice.

Threats can be managed by affirming one's core values and belonging to a group (Proulx et al., 2012). The basic need to belong and to receive recognition (Leary et al., 2006; Fisher et al., 2010) favors coalition formation under stress. Attachment to groups and ideas is pro-social behavior, the purpose of which is to obtain confirmation and protection under duress. Attachment as a system of self-soothing restores the shattered homeostasis of individuals after arousal, anxiety, and anger caused by an eventual mismatch with the environment. Cultural homogeneity, including sacred rituals, are a way to ascertain a sense of being attuned by others (Hart and Sussman, 2011; DiGorcia et al., 2013; Simpson and Karantzas, 2019). Human brains become synchronized by looking at the same object or listening to the same melody (Freeman, 2000).

The need for coherence mediates between negativity bias and political ideologies: when one can fight, one's sense of control is restored. Monitoring external threats, as conservatives tend to do, may be just the sort of tractable task to be completed in order to confirm one's sense of agency. Conservatives are neither fearful nor unhappy, since they are able to deal with dangers externally instead of rationalizing away their immediate emotions at the cost of their balance and satisfaction; cognitive closure makes life less complicated (Hibbing et al., 2014b, p. 337–341). Nationalist populism has been able to turn individual anxieties into a collective fight vis-à-vis clear-cut threats and thus confer a sense of self-efficacy to its supporters instead of a sense of helpless victimization (Bourke, 2005, p. 189–192). Trump promised to do something spectacular immediately, instead of analyzing and ruminating (Lilla, 2016). Marine Le Pen vowed that her first measure as president would be to “re-establish real borders” for France. The Brexiteers won by promising just the same (Krastev, 2017).

The desire to close national borders encompasses both the real attempts to seize control of what happens to oneself and the protective fantasies of fusion of the individual body with the body of the national state. This fantasy of being a part of a greater entity is not pathological as such but a precondition for basic trust to assert one's individual autonomy and to balance the need to belong, on one hand, with individual differentiation, on the other (Blatt and Levy, 2003). Identity groups are experienced as if they were caregivers and described using family vocabulary (“fatherland,” “mother Russia,” “children of France”). Nations can be imagined but they have been able to confer meaning and orientation (Anderson, 2006; Smith, 2010).

A threat against one's self-esteem is expanded to encompass national or western culture as an entity (Dervin, 2015; Nagle, 2017). “Making America great again” promised to ground-losing white males that they could again become great as a part of their large identity group. When collective social identities are defended against perceived disrespect, collective pride suppresses individual shame (Fisher et al., 2010; DeScioli, 2016; Jasko et al., 2017). Large groups establish their identities in counterpoint to those who oppose them and whom they oppose together. Splitting apart and the projection of good and bad do not allow for concern or remorse (Alford, 1989, p. 83–103; Volkan, 1988). This feature matches the conservative lack of neuroticism. The need to remain immune to overwhelming stimulation can override sophistication. In hostile competition, insensitive but hyper-vigilant, risk-taking men succeed: they do not internalize conflicts, empathize or experience shame (Nail et al., 2009; Del Giudice et al., 2013).

During economic advances and increasing divisions of labor, individuals have optimally adapted by means of social skills, empathy, tolerance, and universal care. Individuating features, differentiating interests and the need for self-actualization as a person can prevail, as people feel safe and can free their mental capacity from survival concerns. Chronically threatened people lose cognitive capacities that are normally available to them (Inglehart and Weltzel, 2005; Mullainathan and Shafir, 2013; Weltzel, 2013). High stress reactivity has been associated with a decrease in the more recently evolved cognitive capacity to regulate social and emotional responses (Flinn et al., 2013, p. 110). Creative individuals with a sensitive HPA axis can flourish only when they do not experience threats (Del Giudice et al., 2013).

Only when the survival is guaranteed, people can afford investment in future and be open to new chances. Affluence promotes time discounting, self-control, optimism, cognitive exploration, and social trust (Baumard, 2018). Scarcity of resources seems to bring to the fore conservative virtues that support group cohesion (ingroup solidarity, authority, tradition, purity) (Haidt and Graham, 2007; Graham et al., 2009; Sinn and Hayes, 2017). The evolutionary disposition for coalition formation may benefit conservatives in so far as they become alert to threats from outgroups and rather insensitive to threats posed by impersonal, complex processes, like the climate crisis (Hibbing et al., 2014a).

When threated, groups of people can behave like beehives and try to forget their differentiating interests. To be consistent with their identity, people can sacrifice their economic self-interest and self-expression in favor of tradition, authority, and purity of the group (Haidt, 2012; Kesebir, 2012). Such behavior is not necessarily irrational, since both economic gains and belonging to a group can be seen as motivated by a pursuit of control. Evolutionary psychology points to a gender difference: while women gain comfort from giving and receiving support in long-term personal relationships, men are more prone to think in terms of competing coalitions (Belsky, 2012).

Defensive reactions against unpredictable conditions and offensive actions to gain advantages at the cost of others become blurred in coalitional thinking. Social threats against the *status quo*, so important to right-wing authoritarians, and threats against the privileged position of one's nation, crucial for the social-dominance-oriented, lead both groups to react against the porosity of national borders. People high in RWA believe that the world is dangerous, while the metaphor for those high in SDO is that the world is a competitive jungle. They focus on external dominance over other coalitions (Duckitt and Sibley, 2010; Federico et al., 2013). This shared sensibility concerning coalitional threats favors cooperation between the disrupters of status quo, the competitive egoists, and the disrupted, the risk-averse conservatives.

However, in predicting hostility, RWAs and SDOs differ from each other (Sinn and Hayes, 2018). Theories of the all-encompassing negativity bias (Hibbing et al., 2014a), of a group-binding morality (Graham et al., 2009) or of a general resistance to social changes (Jost et al., 2003) do not catch this. The coalitional mindset as such does not predispose people to coalitional hostility. Positive attachment to one's nation does not necessarily lead to retaliatory hostility against outgroups and a heightened sensitivity to offenses against collective self-worth of one's ingroup: research on Brexit has shown that national identification/attachment (confident self-esteem independent of others' opinions) did not predict the referendum vote, after the collective narcissism (defined as vulnerable self-esteem dependent on comparison and external recognition) was controlled for (De Zavala et al., 2017a,b). Outgroup hostility is evoked first and foremost by status threat (Gidgron and Hall, 2017). Collective narcissism seems to coincide rather with insecure ingroup attachment which sensitizes people to any perceived disrespect (De Zavala et al., 2017b). Obviously, RWAs take to hostile comparisons when they are threatened with loss, while SDOs in their competiveness might always think in terms of zero-sum games (Duckitt, 2006).

Attachment to a group as mechanisms of relieving anxiety is available to everyone but coalition formation with its large-group dynamics favors the right-wing way to detect and manage threats.

“Feeling good” in one's national identification may refer to the experienced protection offered by one's national ingroup. Feeling good as well as a sense of coherence are closely related to a secure status (Gidgron and Hall, 2017). But the possibility of “feeling not so good” before becoming nationally attached, and the possible reasons for uneasiness, have not been covered in the studies on national attachment because attachment is taken as something permanent. National narcissism, too, could be interpreted as a grandiose posture against vulnerability taken after perceived humiliations, not as a pre-existing, malignant and dominant personality trait in the population (cf. Richards, 2018). German retaliatory narcissism has been interpreted as a defense following loss of self-worth after WWI: democratic elites were unable to handle this narcissistic wound but Hitler offered identification with strength (Kohut, 1978/1985, p. 81–94). If hostile narcissism is understood as a way to cope with social comparisons, it becomes historically contextualized instead of remaining a static classification.

## Are Security-Seekers and Disrupters Destined to be Allies?

The increased interest in “situational authoritarianism” among researchers reflects a search for a common human denominator in the midst of the current culture wars. If various protesters could see economic concerns from the same angle, they could cease to imagine each other as “forbiddingly alien and other” (Nussbaum, 1997, p. 85), and cooperate against moneyed interests despite their principled antagonisms in cultural issues. In that case, the populist rhetoric pitting common men against “corrupt” elites (Mudde, 2015; Bos et al., 2018) would possibly increase support for redistribution (Arikan and Cekecioglu, 2019). “Authoritarian neoliberalism,” conceived as redistribution up by mobilizing aggressions down (Streeck, 2015; Bruff and Tansel, 2018, 2019) would fade as the likeliest outcome of the next long-lasting economic crisis.

The challenging question is whether cultural conservatives and economic conservatives can ever oppose each other. Farmers and small entrepreneurs surely have reacted against capitalist land-grabbing, debt slavery and “unfair” pricing to recover what has been rightfully theirs, but have also supported established elites, depending on the threats to their way of life and alliances with other strata (Moore, 1993, p. 92–110, 477–482, 298; Mann, 2012, p. 650–651, 695–718).

Adherence to traditional rules and discipline are the core of cultural conservatism, while economic conservatism is defined by the acceptance of hierarchy, inequality, and intergroup dominance (Altemeyer, 1981; Duckitt and Sibley, 2009, 2010). Since cultural conservatives and economic conservatives are motivated by different concerns, their reactions to economic losses should differ, too, despite their unifying ingroup preference. Nevertheless, normative attitudes concerning groups and families predict economic conservatism as well, and the motive to reduce uncertainty makes even less-advantaged people accept inequality (Thorisdottir et al., 2007, p. 179; Hibbing et al., 2014a, p. 301, 305; Federico and Malka, 2018, p. 6).

From the point of view of political psychology, the unconditional endorsement of private business among RWAs can be explained either as an ontological view of life as competition or as identifying with the system. Trump voters criticized global competition but identified more with American capitalism (Adevedo et al., 2017). They could imagine themselves as entrepreneurs realizing American dream but felt disadvantaged in the meritocratic competition of global elites (Markovits, 2019). Submissive, order-loving persons try to accept the fairness of the world that actually disfavors them. If they can only can see natural or divine order behind actual injustices, hope is sustained. Identification with the established order can and often does override even realistic pessimism about one's chances in that order: false consciousness is not even needed, the search for emotional security by compliance is enough (Thorisdottir et al., 2009; Landau et al., 2015; van der Toorn et al., 2015; Jost, 2017; Jost et al., 2017).

For liberal researchers themselves, one way to save their optimist conception of the innate nature of people has been the redefinition of conservative ingroup morality as altruism. Haidt has tried to make conservatives more familiar in the eyes of liberals by emphasizing their moral commitment to other people in contrast to egoists, be they self-expressive liberals or profit-seeking businessmen. Sinn and Hayes (2017), on the other hand, deny the allegedly broader scope of conservative morality in comparison with alleged liberal “self-actualizers,” emphasizing instead the all-encompassing care evident in liberals and the shared ingroup preference of SDOs and RWAs.

Those whose motivation to care and to be fair is universal, are sensitive to exploitation within groups. Individualizing or universalizing motivation loads negatively against a social-dominance orientation. Liberals can sacrifice individual autonomy for the common good defined universally as environmental conservation, while the authoritarian emphasis on the common good, the social coherence of the ingroup, is connected with outgroup antagonism (including deviating groups within) (Sinn and Hayes, 2017, 2018). Universal morals transcending ingroups and self-expression became conceivable only after the violence of pre-statist societies was eliminated, the need for close-knit ingroups as protectors lessened and affluence allowed individuals personal choices (Berggren and Trägårdh, 2006/2015; Newson and Richerson, 2009; Hruschka and Henrich, 2013; Siedentop, 2014; Sinn and Hayes, 2017).

The inner tension among conservatives between egoist disrupters and altruistic community builders is turned into energizing coalitional synergy: when people defend themselves by supporting ingroup hierarchies, they become less sensitive to exploitation by leading group-members. The evolutionary default setting, the ingroup preference uniting SDOs and RWAs, makes system justification also a rational choice for RWA. By supporting exploitative leaders, cooperating moralists can enjoy the fruits of group-based dominance (Sinn and Hayes, 2018, p. 1124–1126). SDOs can be imagined as alpha males, eager to achieve dominant positions in hierarchical ingroups (Liddle et al., 2012). Dominant alphas are motivated by the lion's share of the booty they get when some outgroup is beaten (Gavrilets and Fortunato, 2014). Altruistic moralists use exploitative bullies (Volk et al., 2012; Garandeau et al., 2014; Goodboy et al., 2016) as their proxies to carry out morally suspect but necessary decisions. Those who submit themselves to the dominant leaders are attracted by their winner-like habitus.

Males in general are ready to cooperate within their group when facing coalitional competition. A hierarchical coalition can prevent free-riding, which improves its competitiveness compared with egalitarian groups (Sinn and Hayes, 2017, 2018, p. 1124; Friesen et al., 2014). According to Sinn and Hayes, authoritarian morale evolved as an adaptation that reduced competition within ingroups struggling for resources against other coalitions. It operates through kin-detection in dividing large non-kin groups, such as religion or ethnicity, as in us vs. them (Heylen and Pauwels, 2015; Sinn and Hayes, 2017, 2018). Populist right-wingers recommend severe punishment for those who infringe on authority (Mudde, 2015, p. 296). Identification with the dominance exhibited by “strong” leaders may compensate for individual insecurity in the labor market. Strongmen such as Berlusconi, Erdogan, Putin, Bolsonaro, or Trump are justified by the mere facticity of their success.

Due to the shared acceptance of hierarchical order, in addition to the shared ingroup preference, the striking difference in risk tolerance does not drive apart those high in RWA and those high in SDO. The SDOs are insensitive to any kind of risk as well as concern for these the risk-takers themselves or other people. RWAs, on the other hand, are not only wary of cultural changes but are still capable of imagining the economic fall-out of risky business behavior on themselves, as well. Loss aversion and authoritarianism go together but the causal mechanisms between them are far from clear. Paradoxically, the reckless risk-takers are driving risk-averse conformers toward the right: the latter adapt to disruptions by identifying themselves with social order and their supposed superiority as conformers (Asbrock et al., 2017; Johnston and Madson, 2017; Federico and Malka, 2018, p. 6–7; Dörre, 2018).

The common superiority motive gluing together SDOs and RWAs must be able to succumb to the centrifugal forces of family values. The short-term life strategy of SDOs explains why they do not care about sexual restrictions. For RWAs, the “binding” motive suggests prosocial sacrifices of individual interests and sexual freedoms for the group cohesion, while SDOs consequently choose egoist strategy. SDOs only feign cooperation but seek power within the groups at the cost of others (Pratto and Hegarty, 2000; Cross and Fletcher, 2011; Kenrick et al., 2013; McCullough et al., 2013; Petersen et al., 2013; Price et al., 2017; Sinn and Hayes, 2017, 2018). RWAs prefer affectionate socialization in stable families and communities (Duckitt, 2001; Peterson and Zurbriggen, 2010; Sinn and Hayes, 2018). SDOs try to maximize their gains in harsh, low-trust and unpredictable environments, while RWAs attempt to establish niches of reliable reciprocation within the competitive world by means of marital fidelity and religious sanctions against disruptive egoism (Boehm and Flack, 2010; Sinn and Hayes, 2018, p. 1134). Those who score high in SDO, score high only in those RWA themes linked with authoritarian aggression while being indifferent in regard to authoritarian submission and conventionalism.

As to the basic conflict between individual rights and social cohesion, SDOs with their materialist values approach cultural liberals, who prefer individual autonomy and self-regulation to traditions. According to Sinn and Hayes, persons high in SDO differ from liberals in the dimension between self-transgression and self-enhancement. They are more self-promoting than altruistic RWAs, who submit themselves to established authorities and reject both self-direction and universalism. Those high in RWA react with authoritarian aggression to normative deviance as a threat to social order, since their peace of mind depends on defensive prejudices and guaranteed social cohesion (Feldman, 2003, p. 46, 67; Feldman and Stenner, 1997; Duckitt, 2006; Passini, 2017, p. 74–76, 80–84; Sinn and Hayes, 2017).

Dissonances are imaginable between reckless winners and conformists, if dominance motivation for the sake of survival does not prevail. For many submissive authoritarians, Trump, the reckless business tycoon and womanizer, did not behave himself respectably enough. He was more able to appeal to aggressive authoritarians, who are close to being social-dominance-orientated and have low agreeableness (Ludeke et al., 2017). The factor that allows most cultural conservatives to ignore Trump's deviations from the religious code of conduct was—according to explicit statements by evangelical leaders—his imagined role as a warrior king overcoming their externalized enemies in the culture wars (Adams, 2018).

Economic questions borrow their affective power from value-laden issues (Johnston et al., 2017). Mass support for capitalism is mediated through the imagined moral order: rewards and losses are seen as deserved or not (Jost, 2017). Conscientiousness has been linked with capitalism and self-responsibility instead of collective social security provided by the state, but this established connection between conscientiousness and conservatism might presuppose fair rewards for self-responsibility (Fatke, 2017). Unfair rewards and punishment could drive hard-working people from the winners of financial speculation, who obviously do not reciprocate in their games (Sinn and Hayes, 2018, p. 231–232, 234). A striking imbalance of gains should also weaken such system justification that is based on the expected coalitional advantages of submission to dominant persons, and strengthen the impact of the phylogenetic disposition of fairness (Van Vugt et al., 2008; Jost, 2017; Starmans et al., 2017). Conscientious people who have fulfilled their duties, react to the eventual loss of income more than careless spenders. Unemployment prevents the conscientious from building up property as a safety valve and shatters the meaning of life. It can cause depression, since the conscientious blame themselves for the losses instead of the conditions of life. If the meaningful relationship between their efforts and the rewards has been broken, men in particular let go; they become irritable and can give up job-seeking (Boyce et al., 2010, 2013, 2015, 2016). In the U.S., white-collars are employed more on the basis of the personal impression they make. If they are rejected, they feel flawed as persons (Sharone, 2014).

Stress experienced by the conscientious is always explained by a real or possible loss of their economic position, their status, their relationships, their beliefs, or their self-esteem (Hobfoll, 1989). The search for confirmation of shattered selves (De Botton, 2005, p. 3–7) may also explain, why conscientious people can react with status enhancement (Gidgron and Hall, 2017). To recruit cultural conservatives to support disruptive economics, political entrepreneurs bundle together contradictory themes by framing economic issues with moral ideals (“freedom,” “family,” “responsibility,” “patriotism”). Emotional frames are charged with anger and disgust that render compromises impossible (Clifford, 2019). According to Federico and Malka (2018), the link between certainty and security needs, on one hand, and conservative political preferences, on the other, is moderated by ideological packages and people's need to follow their party in every respect (identity-expressive motive). In the U.S., even politically passive high authoritarians can satisfy their longing for security and order by supporting redistributive and regulative economic policy. As the catch-all group identification fades, economic interests can have their influence. Even politically active authoritarians can oppose free trade and favor government interventions, if the question is not embedded in the conservative *Weltanschauung*. Politically active citizens, however, defend the party ticket in its entirety (Federico and Malka, 2018, p. 23–28, 31; Johnston, 2017, 2018).

In post-communist societies and among politically less conscious voters in western countries, the preference for economic protection can align with right-wing cultural attitudes, especially among the lower social classes (Lefkofridi et al., 2014; Malka et al., 2017b; Federico and Malka, 2018, p. 33). In such cases, leftist economic policy has represented stability, whereas in western societies, the threat to stability used to come from the left ever since the French revolution. In Eastern Europe, openness to gay marriages has been associated with privatization and austerity programs, while family values have been harnessed by socialist parties (Federico and Malka, 2018, p. 20–21).

According to the meta-analysis by Federico and Malka, the majority of studies show how strong needs for existential security and epistemological certainty (measured as authoritarianism, threat sensitivity, and cognitive closure) go together with cultural conservatism but do not correlate with conservative economic views (e.g., Jost, 2006; Duckitt and Sibley, 2009; Feldman and Johnston, 2014; Malka and Soto, 2015; Johnston et al., 2017; Federico and Malka, 2018, p. 9–18, 27). Logically, the prospect of social backsliding should benefit left-leaning populism (Brown-Ianuzzi et al., 2015).

When leftist politicians can attune themselves to fear of loss and consequent status anxiety (Gidgron and Hall, 2017), they could attract floating voters. The most authoritarian right-wing populists have usually never supported Bernie Sanders in American politics, but Sanders was able to appeal to moderately authoritarian (center-right, “average” authoritarians) and not only to the culturally liberal leftist core. Sanders tried to overcome identity politics and instead emphasize class-based, unifying themes. Among voters in Western Europe, cultural conservatism is often linked with support for redistributive policies (Lefkofridi et al., 2014). Considerable number of center-left populists has been found among the voters for *Alternative für Deutschland (AfD)* (Rothmund et al., 2017). The average voters for the Swedish Democrats in 2018 had suffered economic losses since 2006 and felt the insecurity of working life (Jilani, 2018). Among those who voted for UKIP in Britain to support Brexit, a large group opposed both overwhelming cultural changes and economic insecurity (Harper and Hogue, 2017). During the economic recession since 2008, right-wing populist parties adopted protectionist and interventionist themes able to attract authoritarians on the left from social democrat parties (Mudde, 2013; Lefkofridi and Michel, 2016). Marine Le Pen has been courting the “yellow vest” protesters, evidently airing economic frustration and concerns about the growing inequality under globalization.

Evidence of party interchangeability indicates how cultural conservatives can be moved by economic insecurity as well as by coalitional threats, and how people from the left can at least occasionally seek security by means of cultural closure. Using survey data from 99 nations, Malka, Lelkes, and Soto found not only that right-left attitude organization is uncommon, but that it is more common for culturally and economically right-wing attitudes to correlate negatively with each other, an attitude structure reflecting a contrast between desires for cultural and economic protection vs. freedom. The class of freedom-seekers consists of those who are both economically neoliberal and culturally progressive, whereas the group of protection-seekers consists of those who favor wealth redistribution and cultural conservatism (Malka et al., 2017a,b).

Despite the evidence of widespread risk-aversion, the combination of conservative cultural attitudes and a leftist economic policy remains underrepresented in party politics in Europe (Lefkofridi et al., 2014, p. 66). Paradoxically, individualization works here for total identification with all-good parties fighting against their all-bad adversaries. The post-materialist values of autonomy and self-actualization emerged from a sufficient level of material welfare (Inglehart and Weltzel, 2005), but expectations for individual identity work have persisted after the fragmentation of universal welfare (Koppetsch, 2010). Strong ties with one's overarching group of reference are needed to counterbalance individualized economic risks and the challenges of identity work. Outrage aroused by symbolic insults against one's collective identity can support the personal coherence of lonely individuals: it confers orientation (Brady et al., 2017; Sasse, 2018). This metapolitical search for the mirroring of one's self (Heatherington and Weiler, 2009) provides an affective impetus for party ideologies. The number of floating voters, who consider issues one by one without any consistent ideology, has decreased (Johnston et al., 2017, p. 6; Federico and Malka, 2018, p. 21; Caprara and Vecchione, 2018, p. 62–65, 75–77). Income levels do no longer differentiate voters' values: Republicans and Democrats are bound together by values concerning social issues, not by class. The most heated ideological cleavage divides rich Democrats or rich Republicans. Class issues and class voting have declined in Western democracies since the 1980s (Ciuk et al., 2018, p. 881–883; Inglehart and Norris, 2017).

Alienation from party politics, evident in Europe during the 1990s, worries established, interest-based parties but not populist parties fueled by identity motivation. The U.S. Republican Party has benefited from the overlap of social identities (white, male, protestant) with party affiliation more than the Democratic Party, which consists of overtly diverse subgroups. The GOP functions more than Democrats as a tribe that monitors its symbolic purity (Federico and Malka, 2018; Mason and Wronski, 2018, p. 29).

Experiential openness is linked with easy everyday orientation and the management of life. If the environment is not predictable, and biographical self-experience not continuous, the mind's closing down can be expected depending on both individual dispositions and the perceived threats to one's position. Ontological security presupposes not only a continuous and consistent view of the world but fair social institutions, as well (Giddens, 1991; Kinnvall and Mitzen, 2017, p. 37, 91–92; Harper and Hogue, 2017). Uncompromising values in politics establish epistemological security. RWAs are capable of recognizing economic injustice but their need for security is moderated by their conception of just order.

National populists have succeeded by embedding economic security themes in the ascribed group identity. Front National voters in France have been worried about the future of their way of life, not only their standard of living (Reynié, 2013, p. 37–47, 100–132). In Germany, *AfD* is now more popular than the traditional Social Democratic Party among unskilled and unionized workers. It has been able to turn class struggle into an interethnic competition over welfare redistribution and has labeled traditional trade union leaders as internationalist class traitors. Identity politics guarantees respectability by classifying even low-paid natives, by definition, among hard-working and middle-class Germans, while defining immigrants as lazy (Dörre, 2018). In its economic policy, the *AfD* endorses neoliberalism within national states but favors protective borders. In their comparative analysis of euro-skeptic parties, Slobodian and Plehwe (2018) warn against any premature contrasting of protectionist populists against neoliberal globalists.

Identity politics unite the right wing, whereas it divides the groups on the left along ethnic, cultural, and gender lines. Leftist parties lose the votes of white males by focusing only on women and immigrants while classifying all white males among the privileged. In the alt-right net forums, the left and liberals are routinely included among overbearing global elites. These forums are responding to leftist identity politics with the identity politics of white males (Hawley, 2017; Haider, 2018). Class-based solidarity may be possible, if leftist parties can offer ontological security by means of a convincing economic program and include even white males into its conception of historical progress.

## Discussion and Caveats

Declining economic security and cultural changes amount to status threat: threatened people no longer feel themselves as fully recognized, competent members of their society (Gidgron and Hall, 2017; Fukuyama, 2018). Individual dispositions still matter for political choices, but experiences of economic insecurity drive more voters than 30 years ago toward survival values and away from postmaterialist liberalism (Inglehart and Norris, 2017).

The subjective sense of security can be improved by attachment to group and ideologies. Attachment to groups as prosocial behavior is triggered by perceived threats. Belonging to a group confirms one's individual sense of coherence. Because large groups create their identity in opposition to other groups, a coalitional mindset (especially prevalent among men) is thereby activated. This mindset moves in the same direction as the conservative negativity bias, the sensitivity to external threats by other groups (instead of abstract threats such as economic injustice and environment degradation). Longing for order and orientation may favor right-wing mobilization, while liberal features such as openness, tolerance for ambiguity, and universalism prevent the formation of coherent coalitions. In particular, alt-right trolls externalize and blame their targets without strict moral self-scrutiny, while overtly self-reflective liberals become easily hindered when criticized for being too privileged to speak for vulnerable groups. While empathy and sensitivity have been the best adaptations to complex interdependencies in progressing societies, a lack of both empathy and a sense of guilt may offer a competitive edge for individuals and groups struggling for scarce resources.

The left and liberals are in no way immune to tribal coalition formation, and they create group cohesion through mutual value affirmation as well. However, for now the tribal way of managing threats serves more the right-wing parties, since it depoliticizes class-based themes or transforms them into ethnic rivalry. Left-wing populism, the Occupy movement for instance, had been fragmented from the beginning by an overemphasis of ascribed group differences being an obstacle to mutual cooperation. Fragmented identity groups are not willing to sacrifice their demands for personal recognition in order to find the lowest common denominator for a class-based coalition of divergent individuals.

Groups are bound by ideologies and values. Cultural conservatism and RWA support group cohesion, and SDO is dovetailed with RWA through outgroup hostility: dominant, egoist leaders are chosen as effective representatives of ingroups for zero-sum resource games. Counter-intuitively, disruptive winners (SDOs) and those who seek protection from status threats (RWAs), are seldom driven apart despite their different economic interests, since enlarged kin detection and system justification glue them together. Exploitation within the ingroup and deviations from the moral code of conduct by leaders do not challenge their legitimacy.

Seeking social confirmation of one's worth within an ingroup can be seen as a universal mechanism for coping with anxiety and not limited to conservatives. The coalitional mindset and evolutionary preparedness against external dangers are ultimate causal factors and do not override class politics on their own. Exploring the causal path between economic crisis and populisms may exceed the boundaries of experimental social psychology and presuppose counterfactual imagination.

Kinship or the tribal system of security is activated only when bureaucratic, politically negotiated security networks of developed countries fail or are expected to fail. Coalitional identity politics supplant class-based parties only when these have become unable to relieve the anxieties of voters. The rise of right-wing populism occurred as late as during the financial crisis since 2008, not during the Washington consensus following the collapse of the Soviet Union and the freeing global trade. First, voters under pressure tried the social democrats but were disappointed, since leftist government could offer no alternative to running just to stay in place. Individuals, companies and countries were caught up in the global acceleration of competition, while at the same time wages and social security were being cut (Mann, 1993/2013; Dörre, 2018; Wagner, 2018). Mass immigration was only the last indication of the loss of sovereignty of national states.

Due to welfare retrenchment, economic risks are individualized. Polarization of the labor market also renders people sensitive to status ranking. The increased emphasis on collective identities counterbalance the possibility of losing everything as individual. Zero-sum games for respect between identity groups have been exacerbated during the long recession. Identity politics operates with the deeply ingrained patterns of the coalitional mind rewarding participating individuals with immediate self-satisfaction for being a part of righteous group. Class coalitions, instead, equate to long-term strategic reasoning. The shortening of the horizon of expectations and the causes of the increased short-term reasoning in the West deserves further studies. During the last few centuries, class consciousness has been often encumbered by ethnic, religious, occupational and sectional divisions (Mann, 2012). While universalism was the main strategy for the working classes and minorities to gain equality in industrialized societies for most of the twentieth century, national and religious particularisms have since then channeled the protest of the excluded.

When economic losses and status threats are compensated for by boosting collective identity, political reaction turns away from global capitalism toward the cultural content of globalization. Cultural liberals have been held accountable for the general weakening of the ontological security that economic liberals have promoted through global race-to-the-bottom wage competition and financial bubbles. Culture-liberal leftist parties demanding status sacrifices from western middle-class have been successfully identified as globalist intruders threatening the achieved advantages of working people. In opposition to them, new right-wing parties willingly support the advantages of the western middle-classes, if these advantages are defined vis à vis outgroups and do not threaten the relative competitiveness of national companies. If left-wing parties try to win back the votes of less educated white males, they should include them into their narrative of historical progress. Being able to project oneself into a positive future can confirm one's need for coherence and may alleviate frustrations (cf. Cloninger, 2011).

The findings of political psychology indicate not only the shift of liberals toward the right when under threat and the activation of situational, centrist authoritarianism, but also the fact that passive conservatives or centrist authoritarians may acknowledge economic injustices and occasionally support leftist policies. Unfair losses may make conscientious conservatives angry, but their anger can be deflected by means of system justification or simply by the suspicion that a possible redistribution of wealth would favor groups other than theirs. In particular, ethnic minorities are easily suspected of being free riders (Alesina et al., 2001; Larsen, 2008; Jensen and Svendsen, 2009; Peterson, 2015; Federico and Malka, 2018, p. 35–36). A permanent underclass in absolute need does not elicit as much redistributive compassion as a sudden fall of middle-class income (Delton et al., 2018, p. 911, 919). To gain majoritarian support, redistributive politics would presuppose universal benefits for the middle classes (Arikan and Cekecioglu, 2019, p. 1114), too, and trust in the reciprocal shouldering of its cost between people in different phases of life. Increasing inequality and growing social distances diminish social trust necessary for redistribution (Gärtner and Prado, 2016).

Issues such as the best way of increase welfare, the role of redistribution and immigration remain divisive in any event, and value choices are not ultimately reducible to psychological dispositions. In order to establish the impact of psychological factors on the attitudes concerning economy or immigration, opinion differences should be measured within psychological classes as well, not only between them.

## Author Contributions

The author confirms being the sole contributor of this work and has approved it for publication.

## Conflict of Interest

The author declares that the research was conducted in the absence of any commercial or financial relationships that could be construed as a potential conflict of interest.
